# Deciphering pore-level precipitation mechanisms

**DOI:** 10.1038/s41598-017-14142-0

**Published:** 2017-10-23

**Authors:** N. I. Prasianakis, E. Curti, G. Kosakowski, J. Poonoosamy, S. V. Churakov

**Affiliations:** 1 Department of Nuclear Energy and Safety, Paul Scherrer Institute, Villigen, Switzerland; 2 Institute of Geological Sciences, University of Bern, Bern, Switzerland

## Abstract

Mineral precipitation and dissolution in aqueous solutions has a significant effect on solute transport and structural properties of porous media. The understanding of the involved physical mechanisms, which cover a large range of spatial and temporal scales, plays a key role in several geochemical and industrial processes. Here, by coupling pore scale reactive transport simulations with classical nucleation theory, we demonstrate how the interplay between homogeneous and heterogeneous precipitation kinetics along with the non-linear dependence on solute concentration affects the evolution of the system. Such phenomena are usually neglected in pure macroscopic modelling. Comprehensive parametric analysis and comparison with laboratory experiments confirm that incorporation of detailed microscale physical processes in the models is compulsory. This sheds light on the inherent coupling mechanisms and bridges the gap between atomistic processes and macroscopic observations.

## Introduction

Interaction between fluids and solids, including the precipitation and the dissolution of minerals from and into aqueous solutions in porous media, has a complex feedback to the solute transport in the aqueous phase itself and vice versa^1,2^. There is clear evidence that the macroscopic properties of the medium depend on the physical and chemical transformations that occur at the micropore scale in a strongly non-linear way. For example the formation of nanometer to micrometer-sized precipitate layers around preexisting mineral grains, dramatically reduces the permeability of the medium, thus affecting the macroscopic flow through it, although the total porosity does not change significantly. Flow alteration has in turn an effect on mineral precipitation-dissolution processes. The result is a fully coupled hydro-geochemical reactive transport process. The involved physical mechanisms play a key role in geochemical processes and industrial applications that span from geothermal energy and oil industry to pharmaceutical products, catalysts and long-term nuclear waste containment^3–6^. The prediction of the evolution of such systems is very challenging and can be assisted by conducting dedicated laboratory experiments along with numerical reactive transport modelling^7–10^.

Macroscopic modelling and simulations use a simplistic description of the dynamic processes that actually take place at the microscale. The true evolution of the reactive surface area as well as the alteration of pore size and topology is usually unknown. The change in permeability and diffusivity of a porous medium, due to reactions, is correlated to a global change of the bulk porosity, using empirical laws with adjustable parameters^11^. When a relevant experiment is modelled using a pure macroscopic reactive transport code, the major macroscopic experimental observations can only be reproduced after fitting the adjustable parameters using the same experimental results that are intended to be modelled^9^.

In the case of macroscopic solvers the porosity changes are simply modelled by applying mass balance equations between the inlet and outlet flow rates of the domain of interest. This averaged porosity is then used in the parametric Kozeny-Carman equation to derive an overall permeability and in the parametric Archie’s law to obtain the effective diffusivity. It is therefore not surprising, that smooth simplified relations frequently fail to predict accurately the temporal evolution of the system. It is the microscopic pore level physics per se that ultimately controls the macroscopic processes. Macroscopic models cannot circumvent the lack of microscopic physical description without empirical tuning to the experimental data. Conversely, power laws and correlations, of the porosity variation effect on the permeability and diffusivity, can be derived from microscopic simulations^12,13^ and further up-scaled for use in macroscopic algorithms.

## Reactive transport laboratory experiment, macroscopic modelling and characterization

**Figure 1:**
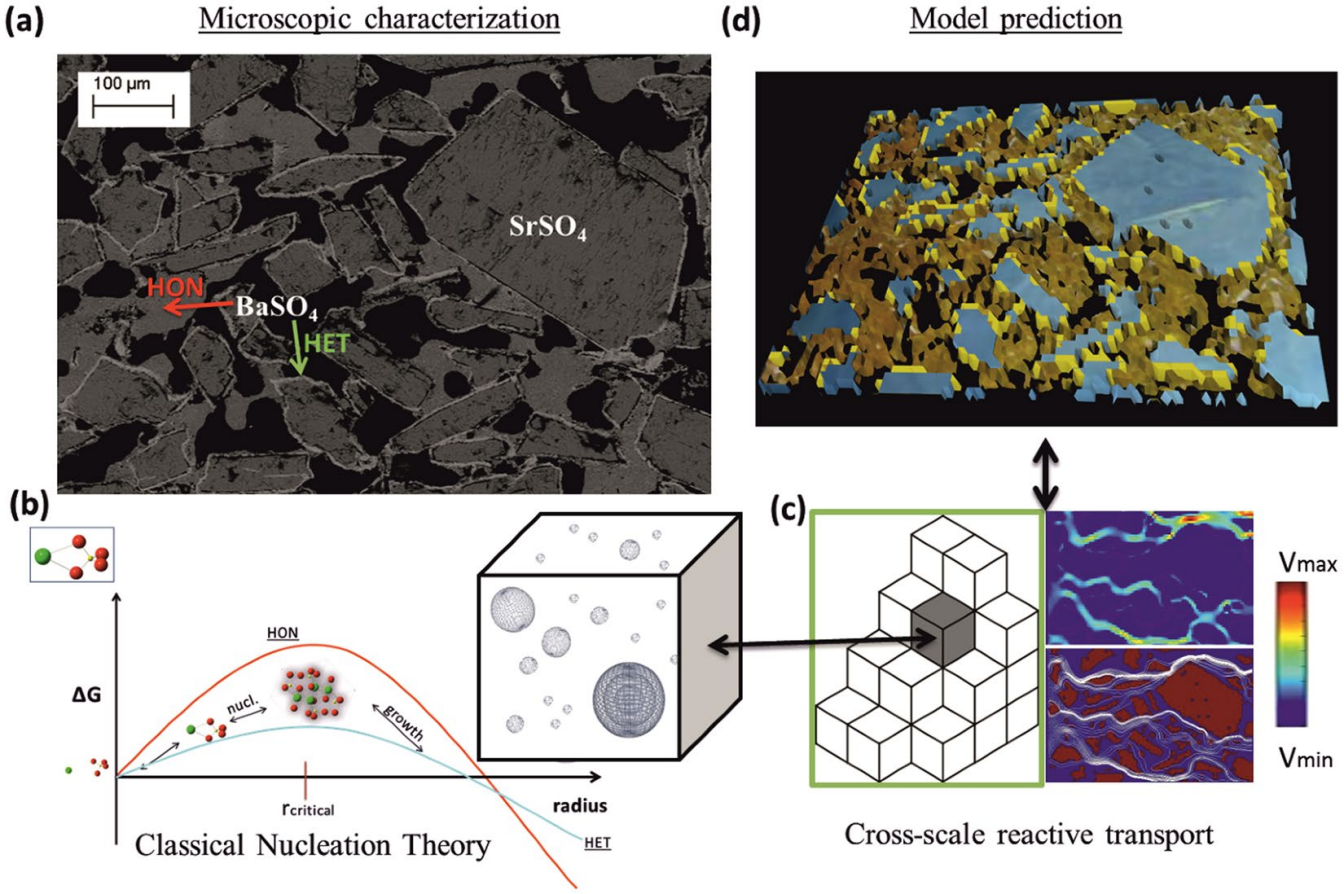
Experiment, underlying processes and modelling. (**a**) BSE-SEM image of the reacted sample with highlighted characteristic features (HON - HET). (**b**) Energy barriers and formation of critical nuclei according to CNT. The computational voxel represents a volume in the bulk where conditions for HON precipitation are met. (**c**) Evolution of HON and HET precipitation is affected by the transport of solutes within the porous medium and vice versa. Colors represent velocity magnitude and white streamlines the preferential flow paths of a generic transport process calculated using the lattice Boltzmann (LB) method. (**d**) Visualization of a model prediction where celestine crystals are depicted in blue and newly formed baryte HON precipitates are in dark yellow, HET in light yellow.

In ref.^9^ we have carried out a reference experiment to elucidate competitive dissolution-precipitation reactions in a granular porous medium. This kind of system is relevant to baryte scale formation in industrial applications at large scale geochemical systems^14^ (hydrocarbon exploration and extraction, geothermal heat extraction, uranium mining, and technologically enhanced naturally occurring radioactive materials-TENORM waste). Further, an exceptionally complete set of thermodynamic and kinetic data is available. The experimental setup, consisted of a strip of reactive celestine (SrSO_4_) with bimodal grain size distribution (<63 µm and 125–400 µm) embedded in a comparatively inert matrix (quartz sand). A barium chloride solution (0.3 M BaCl_2_) was injected into the flow cell, leading to fast dissolution and partial replacement of Celestine (zero ionic strength solubility product constant ${K}_{sp(SrS{O}_{4})}^{0}={10}^{-6.63}$) by the more insoluble baryte (${K}_{sp(BaS{O}_{4})}^{0}={10}^{-9.97}$)^15^ thus modifying the hydraulic and structure properties of the medium (porosity, permeability, connectivity). A subsequent post mortem analysis of the reactor using optical and electron microscopy as well as synchrotron-based micro-X Ray Fluorescence (*µ*-XRF) and micro-X Ray Diffraction (*µ*-XRD) measurements identified two distinct baryte precipitates: i) nano-crystalline baryte filling the pore space and ii) thin baryte overgrowths coating large and medium-sized celestine crystals^16^. The distinction between the two phases was based on the analysis of Debye rings from the μ-XRD patterns after integrating over microscopic sample volumes. The formation of complete Debye rings from tiny volumes of a few μm^3^ indicates that these regions contain a multitude of randomly oriented nanometer-size baryte particles. It should be noted that the absence of Ba-Sr solid solutions is confirmed by the same XRD analysis. This finding is in agreement with thermodynamic calculations and experimental observations^16^ predicting stability of nearly pure baryte and celestine phases^17^ at very low Sr/Ba ratios as used in this experiment. The microscopic characterization shows that two different precipitation mechanisms were operating during the experiments: homogeneous nucleation (HON - nanoparticle aggregate) and heterogeneous nucleation (HET - epitaxially grown rim) both highly dependent on solution metastability (threshold supersaturation). In Fig. 1(a), a Back-Scattered Electron (BSE) microscopy image with the major characteristics of the reacted zone is shown. Large celestine crystals are covered by epitaxially grown baryte layers (HET). Baryte nano-crystalline phase (HON) is also distinguishable and black color corresponds to the remaining open pore-space after passivation of the zone.

The modelling of the experiment using a macroscopic approach could reproduce the evolution of the system only after fitting the kinetic and transport parameters in order to match the experimental results^9^. This level of modelling is not able to explain the presence and formation of two distinct baryte phases nor can provide detailed information of the system evolution, and therefore has limiting predictive capability. This analysis demonstrates the importance of pore-scale understanding of new phase precipitation in porous media. It is clear that in order to improve the predictive capability of the geochemical computational algorithms, such details and effects that span across length and temporal scales have to be incorporated in the modelling.

## Cross scale modelling concept

The basis of our modelling is the simultaneous description of nucleation kinetics, a process that occurs at the atomic scale and of mass transport through realistic porous media. Pore geometry and topology dynamically change due to dissolution and precipitation requiring robust pore-level flow models and solvers. The nucleation mechanisms which lead to the precipitation of ionic solids and particularly of baryte are discussed in detail in ref.^18^ and can be explained based on the Classical Nucleation Theory (CNT). We apply here this concept, in spite of current discussion on the general applicability of CNT^19–21^. As pointed out by ref.^22^, CNT cannot, for example, be applied to carbonate mineral precipitation due to the formation of amorphous precursor phases in this system. CNT is however still a valid concept to explain precipitation of sulfate minerals and particularly baryte, which unlike carbonate minerals does not form allotropic precursors.

In Fig. 1, our cross-scale modelling concept is illustrated. It links the nucleation kinetics and subsequent growth of nuclei that occurs at the atomic level, Fig. 1(b), with the solute concentration subjected to advection and diffusion through the porous structure, Fig. 1(c). In Fig. 1(b) a schematic plot of the total Gibbs free energy (ΔG) for HON and HET is shown as a function of the radius of spherical nuclei. As soon as a critical nucleus is formed and the energetic barrier is exceeded (at the maximum of the ΔG curve) further growth of the nucleus is more favorable than its disaggregation and precipitation starts. For HON nucleation to occur a higher activation barrier compared to HET has to be exceeded thus explaining why homogeneous nucleation requires higher levels of supersaturation to occur. When critical size nuclei are formed precipitation proceeds as growing spheres whose surface area and growth rate is traced throughout the simulation as depicted in the inset of Fig. 1(b). This process is controlled by the larger scale flow and solute features and vice versa. In Fig. 1(c) a generic pore-level flow simulation is depicted where colors represent the velocity magnitude (top) and signify for example the major preferential flow paths which change as the system evolves (the white streamlines in the right-bottom Fig. 1(c)). A model prediction for high saturation index is also visualized in Fig. 1(d) where celestine crystals are depicted in blue and total baryte precipitates are in yellow.

**Figure 2:**
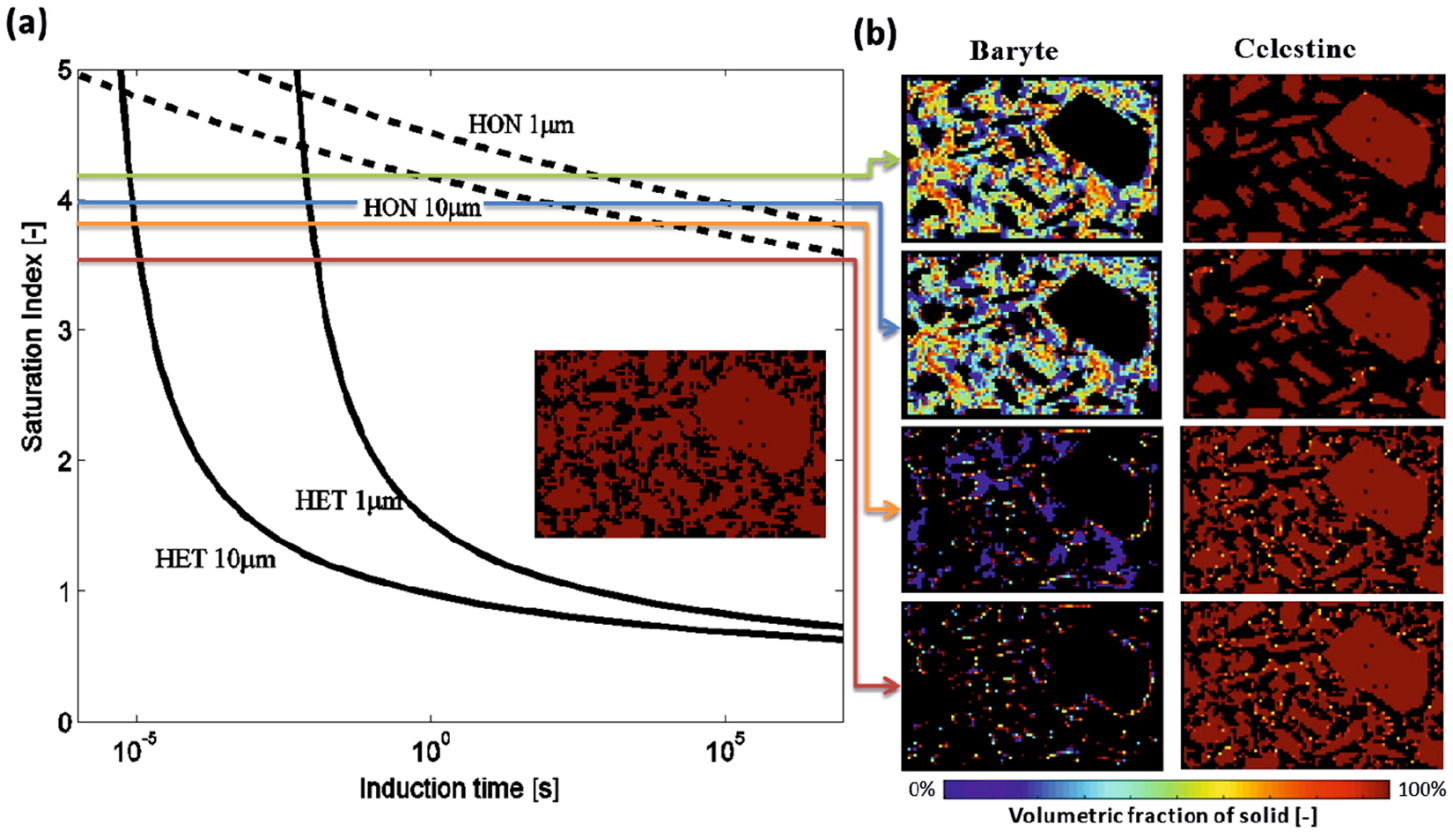
Interplay of precipitation mechanisms. (**a**) Supersaturation - nucleation time diagram including as inset the initial celestine distribution. Saturation index is calculated with respect to baryte. (**b**) Baryte and celestine volumetric fraction after 140 hours of reaction at SI = 4.08, 3.96, 3.8 and 3.6 (top to bottom). Colored arrows represent the predicted evolution of the system for the respective SI, highlighting the interplay of the precipitation mechanisms.

Mineral precipitation via HON from supersaturated solutions is inhibited in porous media compared to precipitation in free solutions^22,23^. This can be explained by a confinement effect of the solution, which makes, according to CNT, formation of critical size BaSO_4_ nuclei more unlikely, the smaller the pore volume^22^. In larger pores the probability of forming supercritical nuclei is higher, and therefore induction times are much shorter. Because of variable pore sizes and non-uniform flow rate distributions, the local concentrations vary in the micrometer range. A Supersaturation—Nucleation—Time (SNT) diagram for homogeneous (HON) and heterogeneous (HET) nucleation optimized for the modelled reactive experiment is plotted in Fig. 2(a) to support the interpretation of the results of Fig. 2(b). In our modelling we dynamically assign, at every point of the domain, the value of its corresponding pore-size. The dependence of induction time on saturation index (SI), for pore sizes of 1 µm and 10 µm is also shown. SI is defined as the decimal logarithm of the ratio of ionic activity product in solution, ${\rm{IAP}}={\alpha }_{B{a}^{2+}}\,{\alpha }_{S{O}_{{4}}^{2-}}$, to the thermodynamic solubility product of baryte, K^0^_*sp*_, i.e. SI = *log*_10_(IAP/K^0^_*sp*_). Throughout the text and wherever mentioned, the saturation index is calculated with respect to baryte.

For the description of simultaneous transport, dissolution and precipitation processes along with the resulting changes of the microscopic pore geometry we applied the lattice Boltzmann method (LB)^12,24–27^. The LB method is a computational technique based on a special discretization of the kinetic Boltzmann equation and provides the necessary fluid dynamic framework (See Methods section). The combination of pore-level transport modelling with CNT can explain the evolution of the considered system and allows predictions under different conditions. For the present problem the discretization grid (lattice) of the porous domain is of the order of 1–10 μm. Sub-micrometer modelling as shown schematically in Fig. 1(b,c), allows to relate the solute transport and concentration with the parameters governing the HON and HET precipitation kinetics, such as the size of critical nuclei, the reactive surface area, the induction time, the local pore-volume and the growth rate. These quantities are locally defined and set the pace for baryte precipitation thus determining the evolution of the system.

## Results and Discussion

The computational domain is setup using the BSE-SEM image of Fig. 1 as a template for the large celestine grains and filling the rest of the space stochastically with small celestine grains, until the initial porosity of the experiment was reached (*ε* = 0.33) as depicted in the inset of Fig. 2(a). The open pores were flooded uniformly with a specified concentration of BaCl_2_ [0.5 M; 0.3 M; 10^−2^ M; 2 × 10^−3^ M] and the solution was instantly replenished yielding a uniform and fixed SI [4.08; 3.96; 3.8; 3.6].

The second case mimics the actual conditions of the experiment where 0.3 M BaCl_2_ solution was constantly supplied via advection^9^. Simulations results are depicted on Fig. 2(b). For SI = 4.08 and SI = 3.96 baryte is precipitating mostly via the HON mechanism and the majority of the dissolved sulfate ions precipitate during the production of the nanocrystalline phase. All small celestine crystals are consumed. Further reduction of the SI increases the induction time of HON and thus favors the epitaxial growth of baryte rims (HET). The system then evolves at a slower pace since there is much less surface available compared to the HON case. In the plot of SI = 3.8 HON precipitation is more pronounced at the regions where larger pore-spaces exist (>50 μm). For SI = 3.6 nucleation proceeds strictly via the HET mechanism for the given reaction time.

**Figure 3:**
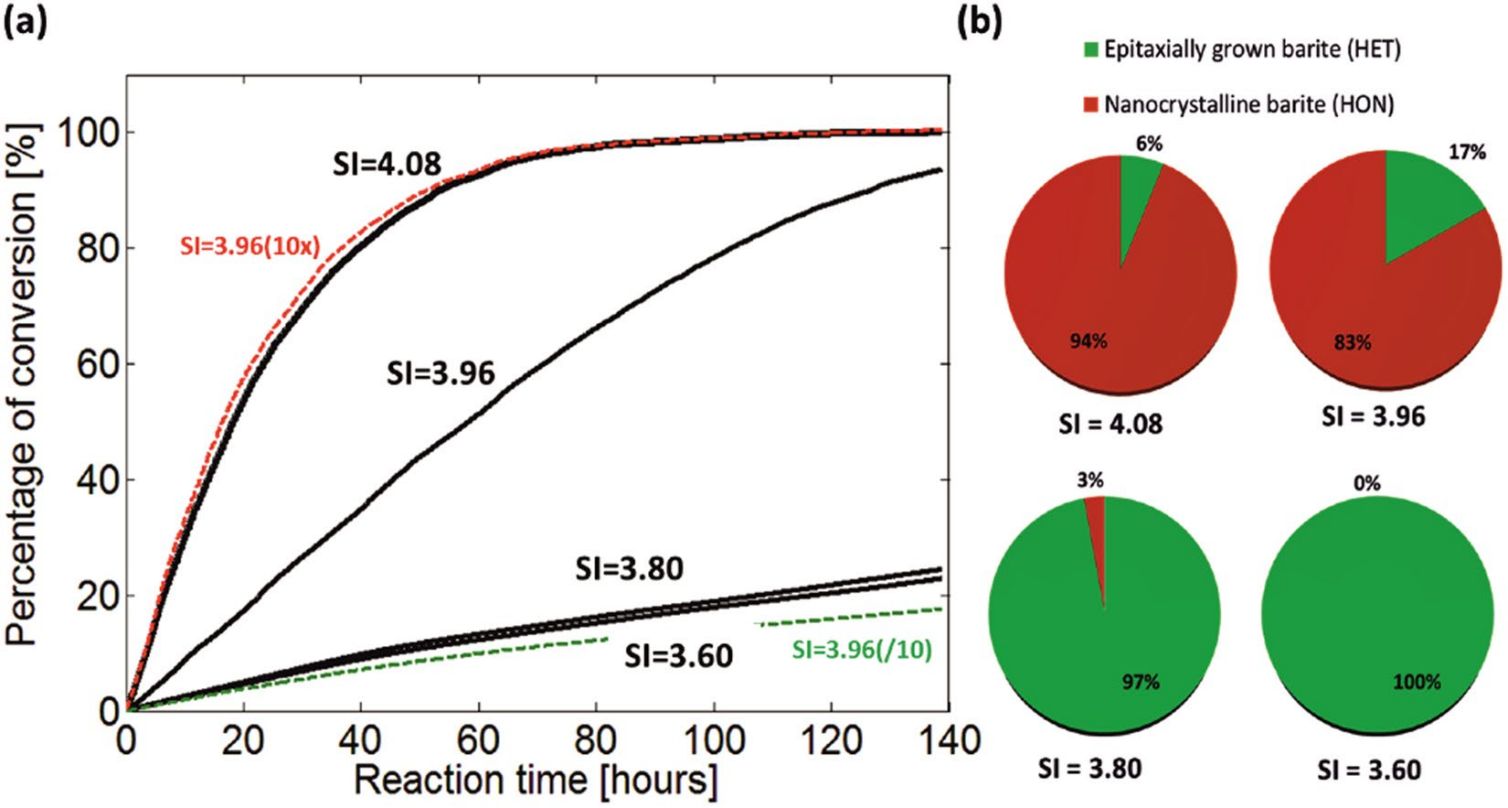
Evolution of precipitation versus time during 140 h. (**a**) Conversion of small celestine crystals to baryte as a function of reaction time (in black) including a nucleation kinetics sensitivity analysis (color), (**b**) partitioning of amounts of epitaxially grown- and nano-crystalline- baryte after 140 h for the different cases.

The evolution of the system after 140 hours of reaction depends non-linearly on the SI and is depicted in Fig. 3(a). The black curves represent the four aforementioned cases. The plotted curves represent the percentage of conversion of small celestine crystals to baryte as a function of reaction time. The complexity of interconnected physical and chemical processes that take place at the pore scale as well as the effect of the degree of suspersaturation on the precipitation pathway is analyzed in Fig. 3(b), in terms of the relative amounts of HON and HET precipitation. We note that porosity changes are proportional to baryte precipitation. Macroscopic models that implement simple analytical relations would fail to predict the effect of these underlying processes.

Precipitation rates measured in the lab in batch experiments often greatly deviate from observations in the field. Reactive surface area, eventual pore topology and flow properties can only partially explain this deviation^10^, thus effects related to the size of the pores must be invoked. The importance and sensitivity of pore size dependent kinetics is illustrated with the dashed colored lines in Fig. 3(a). For the conditions of the experiment where SI = 3.96 we conduct an analysis to highlight the sensitivity of nucleation kinetics with relation to the pore sizes. We perform two additional calculations by keeping all parameters fixed and identical, except for the probability of forming critical nuclei with respect to the pore-size. For that, we increase the probability of forming the critical nuclei in all pores in such a way, that it corresponds to the probability associated to ten times larger (SI = 3.96(10x)- red curve) or ten times smaller pores (SI = 3.96(/10)- green curve). For example, in a 10 μm pore the induction time for the onset of precipitation is tuned as if the respective pore were 100 μm in the first case (10x), and 1μm in the second case (/10). This alteration of the induction times results in a completely different global reaction rate and distinct evolution paths. It demonstrates that kinetic parameters derived from batch experiments cannot always be transferred directly to reactive transport models of porous systems, especially when the average pore size is at the micrometer level. Thermodynamic data acquisition from laboratory experiments must be carefully correlated to the respective pore size distribution.

**Figure 4:**
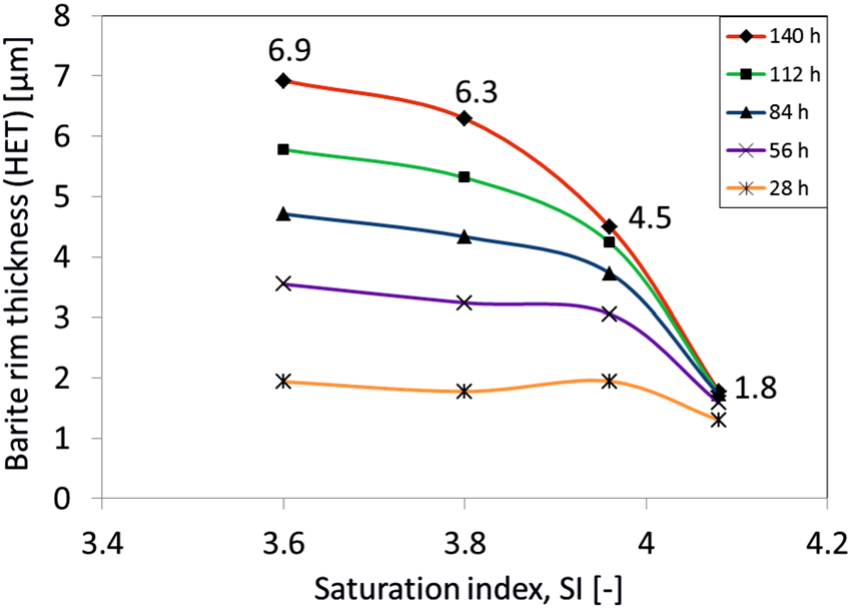
Temporal evolution of the epitaxially grown baryte rim. Higher values of SI result in thinner and slower growth of baryte rims (HET) due to accelerated nano-crystalline formation (HON). Different colors represent the temporal evolution in hours. For SI = 3.96 the average baryte rim thickness is predicted to be *L*_*sim*_ = 4.5 μm and is in very good agreement with the post-mortem analysis of the reactive experiment which measures *L*_*exp*_ = 4.2 μm.

Our modelling allows monitoring also the evolution of the baryte rim thickness and its growth rate which is also strongly dependent on the SI as depicted in Fig. 4. At high SI, the HON mechanism is producing nanocrystalline precipitates at a much faster rate compared to the time needed for sulfate ions to diffuse and reach the growing rims, resulting in thinner baryte layers. Colored lines represent the state of the evolved system after the respective reaction time in hours. In order to compare our predictions with the actual experimental results we compare the epitaxially grown baryte rim thickness after the same reaction time (140 hours). The sample of the BSE-SEM image of Fig. 1(a) was analyzed digitally and the average rim thickness was calculated. The average rim thickness for SI = 3.96 is predicted from our model to be *L*_*sim*_ = 4.5 μm and is in very good agreement with the microscopic post-mortem analysis of the experiment which measured *L*_*exp*_ = 4.2 µm.

## Summary and Outlook

Dissolution and precipitation processes and mass transport phenomena in porous media are coupled at pore level in a complex non-linear way. Macroscopic modelling approaches do not capture microscopic details of this process and therefore face a great difficulty in predicting evolution of such systems^9^. We introduced a cross-scale modelling approach that couples nucleation kinetics based on classical nucleation theory and pore level mass transport on the lattice Boltzmann framework. This coupled description of transport, dissolution and precipitation allows the simulation of complex systems, with a large amount of degrees of freedom at an unprecedented physical detail, provided the necessary parameters are known as in the case of baryte. Quantities such as the size of critical nuclei, the reactive surface area, the induction time, the local pore-volume and the local growth rate have been considered and a good agreement with experimental observations demonstrate the potential of this approach. Moreover, this improves the predictions of the evolution of systems where experiments are too difficult or even impossible, due to limitations in space and time. At the same time it can support and enhance the interpretation of experimental results.

The proposed method has a broad area of industrial and natural sciences applications that range from radioactive scale formation during exploitation of underground energy resources to pollutant dispersion and purification of water^28^. A feature challenge would be to apply this methodology in more complex geochemical systems including solid solutions. Further development would be needed to describe systems where precipitation proceeds along non-classical nucleation pathways.

## Methods

### Classical Nucleation Theory modelling (CNT)

The model developed by Prieto^22^ was used to calculate the Supersaturation-Nucleation-Time (SNT) dynamics for homogeneous (HON) and heterogeneous (HET) nucleation. This model has been integrated in the numerical algorithm in order to calculate the induction time and the kinetics of precipitation at every computational node and at every time step of the simulation. The nucleation rate, J [m^−3 ^s^−1^] varies exponentially with the free energy change ∆*G*_*c*_ associated with the formation of a nucleus of critical size^22^:

1 $$J={\rm{\Gamma }}\,\exp (-\frac{{\rm{\Delta }}{G}_{c}}{kT})$$

where *k* is the Boltzmann constant, *T* is the absolute temperature and Γ a pre-exponential factor. The nucleation energy barrier is highly dependent upon the interfacial tension *σ* and saturation index SI. This barrier is higher for HON compared to HET as shown schematically in the lower part of Fig. 1 of the main text. This explains why HON appears only at relative high values of SI. The exact relation reads:

2 $${\rm{\Delta }}{G}_{c}=\frac{\beta {\nu }^{2}{\sigma }^{3}}{{(kT{\rm{l}}{\rm{n}}({\rm{S}}{\rm{I}}))}^{2}}$$

where *β* is a geometry factor, set to 16.8 for a sphere, *σ* is set to 0.134 J m^−2^ for HON according to^29^, *ν* is the volume of a single spherical BaSO_4_ monomer, and is set to 8.60 × 10^−29^ m^3^ ^22^. For HET, the interfacial tension *σ*_*HET*_, was calculated as a function of the contact angle *θ* between the substrate and the growing mineral, according to^18^:

3 $${\sigma }_{HET}={(0.25(2+\cos ({\rm{\Theta }})){(1-\cos ({\rm{\Theta }}))}^{2})}^{\frac{1}{3}}\,\cdot {\sigma }_{HON}$$

The pre-exponential factor in Eq. 1 represents the rate of attachment of monomers controlled by diffusion and is given as Γ = 2*πZDN*_1_ *N*_0*dc*_, where *D* is the diffusion coefficient of monomers set to 9.3 × 10^−9^ m^2^ s^−1^ and *d*_*c*_ = 4*σv/kTln*(*SI*).

Parameters *N*_0_ and *N*_1_ are concentrations that represent the numbers of monomers per unit volume of fluid and the number of nucleation sites respectively. After^22^ these parameters were set to *N*_0(*HON*)_ = 3.33 · 10^28^ m^−3^ and *N*_0(*HET*)_ = 2.50 · 10^13^ m^−3^. Parameter *N*_1_ depends on the supersaturation which has been evaluated using the Gibbs Energy Minimization Software - GEMS^30^. *Z* is the Zeldovich factor given as $Z={(\frac{{\rm{\Delta }}{G}_{c}}{3\pi kT{({n}_{c})}^{2}})}^{\frac{1}{2}}$, where the number of monomers in the critical nucleus, *n*_*c*_, is given as: ${{\rm{n}}}_{{\rm{c}}}={(\frac{2{\rm{\sigma }}a}{3{\rm{kTln}}({\rm{SI}})})}^{3}$ where *a* is the surface area of a single nucleus. The appearance of many nuclei and their growth are responsible for the breakdown of the solution metastability. This is quantified as the induction time t_i_^18,31^:

4 $${t}_{i}=(\frac{1}{J{V}_{p}})$$

where V_*p*_ is the volume of the local pore under consideration. Note that the implemented algorithm measures and updates the size of the respective pores at every time step, which changes due to dissolution (size increase) or precipitation (size decrease). This is necessary for the correct calculation of the necessary the local induction time needed to pass before the onset of precipitation.

Baryte precipitation rates are calculated according to the empirical kinetic law proposed in ref.^32^. This law was derived from systematic precipitation experiments both in the presence and absence of precipitation inhibitors:

5 $$-\frac{d[Ba]}{dt}=A\times {k}_{\ast }{K}_{sp}^{0}{(\sqrt{{10}^{({\rm{SI}})}}-1)}^{2}$$

where ${k}_{\ast }{K}_{sp}^{0}=8.6\times {10}^{-9}{\rm{mol}}\,{{\rm{m}}}^{-2}\,{{\rm{s}}}^{-1}$, and A [m^2^ m^−3^] is the relative surface area per unit volume. For HON, a sublattice model with respect to the transport solver is implemented as discussed in the main text. The sum of molecules that form the first nucleation clusters are converted to equivalent spheres. The resulting reactive surface area is calculated based on the evolving area of these virtual spherical objects. More than one critical nuclei are allowed to be simultaneously formed, and their growth is traced throughout the simulation (See Fig. 1(b) of main text).

### Lattice Boltzmann modelling details (LB)

Pore-level processes can be simulated with a variety of numerical methods^33–35^. For the modelling of the advection-diffusion and precipitation processes a multi-component LB model is used. The model is composed of a basis fluid medium that recovers the Navier-Stokes equations at the macroscopic limit, plus several passive scalar coupled population sets that simulate the diffusion of ions. The isothermal guided equilibrium nine-velocity model (D2Q9 lattice) of ref.^36^ is selected as the basis model. The discrete velocities of populations *f*_*i*_ for i = 0–8, are *c*_*i*_ = (0, 0) for *i* = 0, *c*_*i*_ = (±1, 0) and (0, ±1) for *i* = 1–4, and *c*_*i*_ = (±1, ±1) for i = 5–8^37^.

The following population-moments correspond to the density of the solution *ρ* and the momentum *j*_*a*_ in the direction *a = x*, *y*:

6 $$\sum _{i=0}^{8}{f}_{i}=\rho ,\quad \sum _{i=0}^{8}{c}_{ia}\,{f}_{i}={j}_{a}$$

The guided equilibrium populations ${f}_{i}^{eq}$ are given in a closed form, where T_0_ = 1/3:

7 $${f}_{i}^{eq}=\rho \prod _{a=x,y}\frac{(2{c}_{ia}^{2}-1)}{{2}^{{c}_{ia}^{2}}}({c}_{ia}^{2}-1+{c}_{ia}{u}_{a}+{u}_{a}^{2}+{T}_{0})$$

The Boltzmann BGK equation is solved: ${\partial }_{t}\,{f}_{i}+{c}_{ia}{\partial }_{a}\,{f}_{i}=-\frac{1}{\tau }({f}_{i}-{f}_{i}^{eq})$, where *τ* is the relaxation parameter and $\mu =\tau \rho {T}_{0}$ is the resulting macroscopic dynamic viscosity. BGK stands for the Bhatnagar-Gross-Krook collision model as depicted in the right hand side of the aforementioned equation and describes the relaxation of populations *f*_*i*_ to their equilibrium state *f*_*i*_^*eq*^ with relaxation time *τ*. For the advection-diffusion reaction equations of the reactive species a D2Q9 model is also implemented. Although the option of a D2Q5 model would be computationally more efficient, the D2Q9 lattice is preferred since the extra diagonal velocities offer a more physical description at the nodes where dissolution and precipitation take place.

The equilibrium populations ${{\xi }_{i}}^{eq}$ for the reactive species ${\xi }_{i}$ are given:

8 $${\xi }_{i}^{eq}={C}_{i}\prod _{\alpha =x,y}\frac{(1-2{c}_{i\alpha }^{2})}{{2}^{{c}_{i\alpha }^{2}}}({c}_{i\alpha }^{2}-1+{c}_{i\alpha }{u}_{\alpha }+{T}_{0}),$$

where ${C}_{i}$ is the concentration of [Ba^+2^],[Sr^+2^], [Cl^−1^] and [SO^−2^_4_] ions and ${u}_{\alpha }$ is obtained from the basis model. The relevant population moment that corresponds to the concentration is:

9 $$\sum _{i=0}^{8}{\xi }_{i}={C}_{i}$$

### Experimental setup

**Figure 5:**
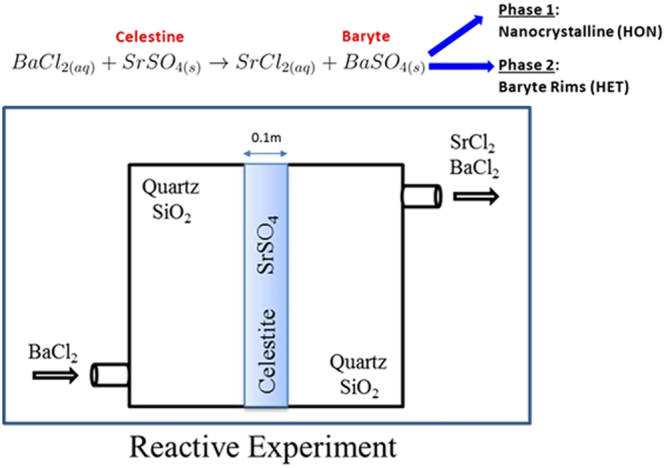
Schematic of the laboratory experiment. A zone of celestine grains (SrSO_4_) is positioned between two layers of quartz (SiO_2_). Barium chloride BaCl_2_ rich solution is injected from the inlet on the left side and causes the dissolution of celestine. Baryte precipitation changes the topology and hydrodynamic properties of the former celestine zone. Post mortem microscopic characterization the reacted zone allows identifying the involved precipitation mechanisms.

The dissolution-precipitation experiments and post-mortem analysis and interpretation are described in detail in refs^9,16^, we therefore give only a summary of the work with details relevant for understanding the modeling. An acrylic flow cell of internal dimensions 10 cm × 10 cm × 1 cm was filled with a reactive porous medium sandwiched between two layers of an inert granular porous media as depicted in Fig. 5. The inert granular medium was a commercial acid washed and calcined quartz sand (SiO_2_) of 99.1% purity. The reactive porous medium was created of naturally occurring celestine from Madagascar which comes in form of polished stones. The natural celestine was analyzed by X-ray diffraction which revealed a celestine content of 99.7% with 0.3% of anhydrous calcium sulfate. The stones were crushed and sieved to give batches of different grain size. The reactive medium was a mixture of grains: 30 wt. % with a size of less than 63 µm and 70 wt. % with a size of 125–400 µm. After compaction of the mixture a porosity of 0.33 ± 0.01 was measured. A stationary flow field was established by injection of a solution saturated with strontium sulphate to prevent strong dissolution of Celestine. This initial flow field, as well as flow field changes during the subsequent reaction experiments were visualized by injection of dye tracers.

For the reactive experiment a solution of 0.3 M barium chloride was injected at a constant flow rate of 20 µL min^−1^, The initial strontium sulfate saturated solution has a lower density as the injected barium chloride solution; therefore at the start of the experiment the density differences enforced a transient flow and transport regime while the injected solution moved along the bottom of the tank. As the barium chloride solution reached the celestine region, the dissolution of celestine and the precipitation of baryte occurred, inducing changes in the pore space topology. We observed a strong decrease in permeability of the reactive layer. These changes were reflected in a rising level of barium chloride on the injection side of the tank which causes the ingress of barium chloride into increasingly higher parts of the reactive layer. The chemical composition of the effluents was measured (Cl^−1^, SO_4_^2−^, Ba^2+^ and Sr^2+^) using ion chromatography. At the end of each experiment, but before dismantling the flow cell, isopropanol was injected in order to stop further chemical reactions. The reacted celestine was found to be solidified into a rectangular block. The reacted medium was dried and impregnated under vacuum with araldite XW936/XW397. An extensive post-mortem analysis of the reacted celestine layer was performed with different techniques including scanning electron microscopy, and synchrotron based X-ray micro-diffraction/micro fluorescence performed at the XAS beamline (Swiss light source).

The governing reaction describes the dissolution of solid celestine crystals and the precipitation of solid baryte. Baryte precipitates via two distinct mechanisms producing nano-crystalline (HON) and epitaxially grown rims (HET):

10 $$BaC{l}_{2(aq)}+SrS{O}_{4(s)}\to SrC{l}_{2(aq)}+BaS{O}_{4(s)}$$

A schematic of the experimental setup can be seen in Fig. 5

## Electronic supplementary material

Supplementary Video 1

Supplementary Video 2

Supplementary Video 3

Supplementary Material
