# Supplementary material for: Deciphering pore-level precipitation mechanisms
Source: Sci Rep. 2017 Oct 23;7:13765. doi: 10.1038/s41598-017-14142-0 (PMC5653867; doi:10.1038/s41598-017-14142-0)
Supplement: Supplementary file 4 — Supplementary Material [file 41598_2017_14142_MOESM4_ESM.doc]

# Supplementary Material

**Deciphering pore-level precipitation mechanisms**

# N. I. Prasianakis^1,*^, E. Curti^1^, G. Kosakowski^1^, J. Poonoosamy^1^, and S. V. Churakov^1,2^

^1^Department of Nuclear Energy and Safety, Paul Scherrer Institute, Villigen, Switzerland

^2^Institute of Geological Sciences, University of Bern, Bern, Switzerland.

^*^Corresponding author: [nikolaos.pr](mailto:nikolaos.prasianakis@psi.ch)[asianakis@psi.ch](mailto:asianakis@psi.ch)

## Supplementary Video 1

Video showing the evolution of celestine dissolution during 140 hours at SI=3.96. Colorcode represents the volumetric fraction of solid

## Supplementary Video 2

Video showing the evolution of baryte precipitation during 140 hours at SI=3.96. Colorcode represents the volumetric fraction of solid

## Supplementary Video 3

Comparison of competitive dissolution-precipitation for three different concentrations: SI is 4.08, 3.96 and 3.6. Left set of movies show the computational domain. Right set of movies are zoomed in representations. Third dimension is the volumetric fraction. Blue corresponds to celestine and yellow to baryte.
